# The educational environment of a traditional public school of medicine in Brazil with the DREEM questionnaire

**DOI:** 10.15694/mep.2019.000101.1

**Published:** 2019-05-02

**Authors:** Débora Alves dos Santos Fernandes, Stella Regina Taquette, Nádia Cristina Pinheiro Rodrigues

**Affiliations:** 1 School of Medicine and Surgery; 2 Faculty of Medical Sciences; 3 National School of Public Health Sérgio Arouca

**Keywords:** Medical Education, Learning Environment, Perceptions of Medical Students, Teaching and learning, DREEM.

## Abstract

This article was migrated. The article was marked as recommended.

**Introduction:** The educational environment (EE) of medical education is the set of factors of a material and affective nature that permeates the tripod formed by the educational institution, by the teacher and by the student. A healthy environment results in quality learning and, consequently, a professional with a higher level of competence and satisfaction.

**Objectives:** To analyze the educational environment of a medical school public university in Brazil in the view of its students and to investigate the presence of association between the sociodemographic variables of the students and their perceptions about the EE.

**Methods:** A cross-sectional observational study with medical students from four different periods of the course, using the questionnaire DREEM and a sociodemographic questionnaire.

**Results:** 210 students participated. Although DREEM’s overall score shows that students have a more positive rather than negative view of EE in general, there was a large discrepancy in results among the groups surveyed, indicating that the more students progress in the medical course, the more negative your view on the educational environment. The perception about the general teaching environment and social relations was negative in the four groups. Female students, white, and those who lived outside Rio de Janeiro before attending in medical school perceived EE more negatively. The perception of EE by students who entered the system of racial or social quotas and those who did not join the quota system was similar.

**Conclusion:** the analysis of the educational environment the students’ dissatisfaction with the traditional Flexnerian models. Low-income, self-declared non-white and female students experience more problems and negative situations in the medical school. That teacher-centered teaching is an unfavorable factor in EE and discourages the progressive intellectual and professional autonomy advocated by the current National Curricular Guidelines of the Brazil.

## Introduction

The Educational Environment (EE) is the set of elements of material and / or affective order that involves and pervades the learner and should be conducive to learning. Learning motivation and perception of EE by the learner, in turn, may be affected by the context and environment in which learning is taking place ( Hutchinson, 2003).

The term “educational environment” (EE) is polysemous and has been used in many different ways. In this research was used the concept of Genn ( Genn, 2001a) in which the EE is defined as any and every context in which teaching and learning take place.

The EE has a direct impact on students’ academic performance, as well as their satisfaction with the educational process ( Miles, Swift and Leinster, 2012). This impact can be evidenced by research carried out in the area of medical education in several countries of diverse continents, such as in Thailand ( Pimparyon *et al.*, 2000) and medical schools in India ( Mayya and Roff, 2004), Chile ( Herrera *et al.*, 2010) and Saudi Arabia ( Al-Kabbaa *et al.*, 2012).

The learning environment is a determining factor of the young doctor’s professional conduct. EE is of great relevance in medical school and curriculum, and is related to student success, satisfaction and success ( Genn, 2001a). The curriculum implemented in the medical teaching institution does not define the EE, however, as well as the faculty and student body, is one of the fundamental factors for the appropriate teaching-learning environment.

At the beginning of the twentieth century there were three medical schools in Brazil ( Gonçalves and Benevides-Pereira, 2009) and the pedagogical model adopted was French academicism and research was influenced by the German school. Successive reforms and time made this model totally replaced by the American, hegemonic in the early nineteenth century. From the Flexner Report ( Flexner, 1910), the curriculum of medical teaching began to meet the Flexnerian model. In 1968, the Minimum Curriculum in the country came into force, according to the current legislation ( Brasil, 1968). However, this curriculum proposal continued to be based on the teaching model of a Flexner course.

Several events since the 1950s have contributed to the debate about reforms in medical education ( Gonçalves and Benevides-Pereira, 2009), movements or proposals that sought to reorganize Brazilian medical education, including discussing the professional that the school should train: a) the creation of the Coordination of Improvement of Higher Education Personnel and the National Research Council to improve higher education and encourage research; b) the actions of the Rockfeller and Kellogg Foundation in support of health projects; c) the incorporation of concepts such as integral medicine, preventive medicine, community medicine and, later, family medicine.

In 2001, the Brazilian government approved the National Curricular Guidelines for Undergraduate Courses in Medicine (NCG) ( Brasil, 2001). The NCG recommended changing the format of medical courses in Brazil, from a mirrored orientation in the disease-centered flexnerian approach to student-centered teaching. In addition, the NCG advocate practices that stimulate a progressive intellectual and professional autonomy of the student, so that they favor the theory-practice articulation and development of the teaching-learning process. In 2014, the Brazilian government instituted new NGC ( Brasil, 2014), encouraging a generalist training of the physician and enabling the implementation of the More Doctors Program ( Brasil, 2013). This program aims to ensure a restructuring of the Unified Health System - Brazil, modifying the EE of the students. The More Doctors Program reshapes the SUS through its three axes of action which are the emergency provision of professionals to regions where there is a lack of doctors, implementation of medical teaching institutions focused on regional health characteristics, and infrastructure through maintenance and construction physics of health services. Universities and medical colleges are tasked to tailor their curricula to NGC and its EE to these new scenarios of theoretical and practical teaching.

Brazilian research in the last decade has shown that the incidence of depression and suicide among medical students is five times higher than in the general population and in other academic groups ( Meleiro, 1998; Santa and Cantilino, 2016) including the prevalence of moderate to severe depression of 60% among medical students ( Vasconcelos *et al.*, 2015).

Investigating the quality of the EE is a fundamental tool for the creation of institutional policies that guarantee motivational teaching and the student’s quality of life, both physically and emotionally. The result of the AE analysis can guide interventions that reduce alarming rates of depression and suicide among medical students in Brazil.

This article is part of a Master’s Dissertation on EE of public and traditional medical school in Brazil and aims to contribute to the improvement of the quality of the medical course, taking into account the academic period where the student is and their sociodemographic characteristics.

It aims to analyze the educational environment of a medical school in the view of its students and to investigate the presence of association between the sociodemographic variables of the students and the general perception of the EE.

Every effort to recognize and improve the EE of a medical school is fundamental to contribute to the improvement and evolution of the educational process itself of medical education ( Genn and Harden, 1986). The continuous pursuit of improving quality from the environment promotes benefits in medical schools, so that they become true learning organizations. Unless medical schools become such organizations, their quality and longevity may be threatened ( Genn, 2001a).

## Methods

This study is a cross-sectional observational study using the Dundee Ready Education Environment Measure (DREEM) questionnaire ( Roff *et al.*, 1997), validated for Brazil in 2003 ( De Oliveira Filho, Vieira and Schonhorst, 2005) and a sociodemographic questionnaire, both applied in a single moment for each participant.

The DREEM, the chosen educational evaluation instrument, had its validity and reliability confirmed by several studies and has proved to be more comprehensive and multifunctional ( Soemantri, Herrera and Riquelme, 2010). It is an instrument originally designed to be a universal, applied in various parts of the world, and serves as a basis for the development of more particularized instruments. It was developed at the University of Dundee, Scotland, based on the opinion of students and teachers and submitted to the appreciation of 48 mid-career teachers from 22 different countries ( Roff *et al.*, 1997). This instrument is composed of 50 statements, which reveal different perceptions of the educational environment, in relation to which students manifest themselves through Likert scale. The analysis and interpretation of the results of the DREEM scores was performed according to the practical use guide proposed by Sean Mcaleer and Sue Roff of the University of Dundee, United Kingdom ( McAller S, 2001).

The questionnaires were applied in classrooms and in medical practice environments of the university hospital of a public university of Medicine, located in the State of Rio de Janeiro, Brazil.

The institution where the research was carried out is one of the oldest in Brazil and carried out its first curricular reform in 1999, based on the Brazilian minimum curriculum of 1968 and the Flexnerian model. In 2014, implemented a new curriculum reform, based on the NGC of 2001. Since then, the teaching is changing and it is necessary to verify if these changes in the curriculum reach what the Brazilian guidelines advocate and if the institution has an AE suitable for teaching -learning.

Data collection took place in July 2017 (for 1st, 4th and 9th grade students) and November 2017 (for the 12th grade), and was carried out by the first author. We included students of both sexes, of any age, enrolled and studying medicine at the institution, who signed the Free and Informed Consent Term.

In the first period the students attend the basic cycle of the medical course. In the fourth and ninth periods they attend the clinical cycle, and in the twelfth period they attend the boarding school. Data from the twelfth period were collected two weeks prior to the graduation of physicians in the practice settings at the university hospital, specifically in the clinic and clinic wards and outpatient clinics, in Clinical Surgery, in Clinical and Surgical Specialties (Ophthalmology, Otorhinolaryngology, Traumato-orthopedics, Anesthesiology, Urology, Dermatology), Pediatrics and Gynecology and Obstetrics.

**Table 1.** Educational profile of the groups participating in the study

**Academic period**	**Time at the EMC**	**Teaching cycle**	**Curricular reform**	**Theoretical classes** **(preceptors)**	**Practice scenarios**
1st period	4 months	Basic	Yes	school teachers	Basic Health Center
4th period	2 years	Clinical	Yes	school teachers	University Hospital
9th period	4 years and 6 months	Clinical	No	school teachers	University Hospital
12th period	6 years	Internship	No	school teachers	University Hospital and other public hospitals in Rio de Janeiro

In the statistical analysis of the information collected, in order to compare the scores of the five dimensions and the overall DREEM score between the groups, measures of central tendency and dispersion (mean and standard deviation) were used for continuous variables and frequency distributions (percentage) for the categorical variables.

The data were entered in Microsoft Excel 2016 for Windows spreadsheets and statistical analyzes were performed using the statistical software R version i386 3.5.0 and R Studio, through hypothesis test (Student t) and analysis of variance (ANOVA).

The research was submitted to the Ethics and Research Committee’s appreciation and began after its approval through of the number 1.845.082 and the Presentation Certificate for Ethical Appreciation (CAE) number 60942116.4.0000.5258.

## Results/Analysis

The results obtained are described according to the characteristics of the study population, with the five perceptions (dimensions) and the overall score, for each group as well as for the total of the research participants.

The DREEM questionnaire was well accepted by the students, since all the students that met the inclusion criteria participated in the research. This good acceptance was also found in other studies (22-24).

Two hundred and ten students participated, being 80 of the first period, 43 of the fourth period, 44 of the ninth period and 43 of the twelfth period. The population base of the study comprises 800 students enrolled in the medical school at the time of data collection. Two hundred and ten students fulfilled the inclusion criteria and participated in the research. These comprise 72% of the total students enrolled in the study periods studied and 22% of all students enrolled in the EMC at the time of the survey. The mean age of the students studied was 23 years, the majority female (59%) and the majority self-declared white (61%).

In order to have an overview of the studied population, the main sociodemographic characteristics are described in table 2.

**Table 2.** Socio-demographic characteristics of the studied population

Variable	**População**
**Sex** Female Male	107 (51%) 103 (49%)
**Color** White Not white	124 (59%) 83 (41%)
**Age** < 2020-22 23-25 >26	44 (21%) 60 (28.5%) 46 (22%) 59 (28.5%)
**Monthly value for self-support of the student** Up to R$ 1.250 (approximately US$ 350) More than R$ 1.250 (approximately US$ 350)	83 (40.5%) 125 (59.5%)
**Student work** Does not work works	183 (87%) 27 (13%)
**Middle of Medical School entry** SiSU - ENEM Social quotas	166 (79%) 41 (21%)

### Sociodemographic profile of the study population and DREEM score

Among the different perceptions, the most negative view was revealed by students of the 12th period, by female students and older than 26 years. Table 3 refers to the data grouped into categories and analyzed according to the total DREEM score.

**Table 3.** Sociodemographic variables and total DREEM score

Variable	N (%)	Mean DREEM (SD)	P-value
**School year** * 1st period 4th period 9th period 12th period	56 (32%) 38 (21.7%) 38 (21.7%) 43 (24.6%)	152.04 (16.98) 147.16 (14.46) 138.47 (18.03) 99.42 (6.90)	<0.0001
Sex * Female Male	89 (50.8%) 86 (49.2%)	128.51 (25.26) 141.93 (24.34)	0.0004
**Color** • White Not white	105 (60.6%) 68 (39.4%)	132.47 (26.97) 140.09 (22.50)	0.05
Age * < 20 20-22 23-25 >26	31 (17.8%) 52 (29.8%) 38 (21.8%) 53 (30.6%)	152.39 (16.69) 148.13 (16.67) 132 (22.45) 114.02 (24.30)	<0.0001
Monthly value for self-support of the student * Up to R$ 1.250(approximately US$ 350) More than R$ 1.250(approximately US$ 350)	69 (39.9%) 104 (60.1%)	141.67 (23.35) 130.38 (26.30)	0.07
**Student work** Does not work works	152 (86.9%) 23 (13.1%)	135.06 (25.70) 135.39 (25.81)	0.95
**Middle of Medical School entry** SiSU - ENEM Social quotas	142 (82%) 31 (18%)	133.56 (25.66) 142 (25.60)	0.41

Note 1: * **P-value** < 0.05 by ANOVA

Note 2: • **P-value** < 0.10 by ANOVA

Note 3: SD: Standard deviation

**Figure 1.**
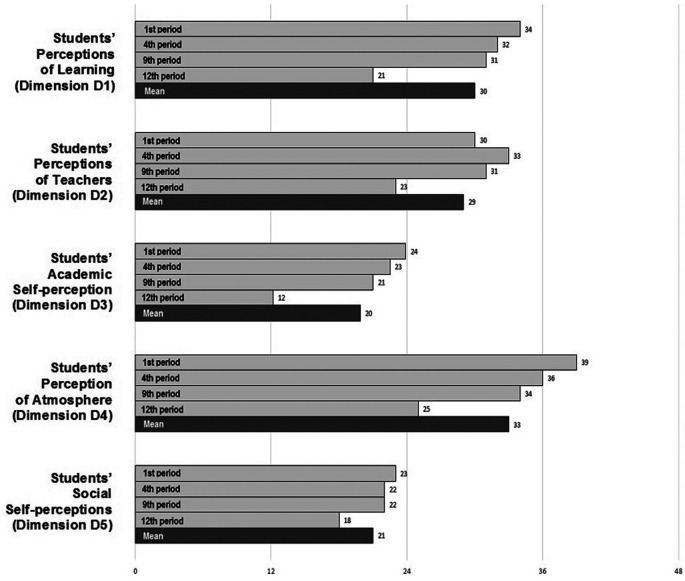
Comparative description of the perceptions score: interpretation of the score of the academic periods and the average score of each period

The overall EE was perceived as excellent for the first period (“excellent” - 152.0) and more positive than negative for the fourth and ninth periods (“more positive than negative” - both 138.5). The twelfth period reveals a negative view of the EA (“plenty of problems” - 99.4). The mean of the four groups revealed a general positive rather than negative overall (“more positive than negative” - 132.1) ( Figure 1).

White students aged 12 and over who use more than R$ 1.250 (approximately US$ 350) for their own living have revealed a more negative view of EC. There was no statistically significant variation among students in relation to work or means of university admission.

### Students’ Perceptions of Learning (Dimension D1)

The score found revealed that the first, fourth and ninth periods have a positive perception of learning (“a more positive perception” - 34.32, 32.07 and 30.52 respectively). The twelfth period, which revealed a negative perception of teaching (“teaching is viewed negatively” - 21.49) ( Figure 1). This difference in scores between the periods was statistically significant. The mean of the four groups was 29.6, revealing a more positive overall perception of learning (P-value <0.05).

### Students’ Perceptions of Teachers (Dimension D2)

The scores found in the four groups revealed a good overall perception of the “right-handed” preceptors, with the mean among the four groups being 29.47. The score for the 12th period is the lowest, 23.19 (P-value <0.05) ( Figure 1).

### Students’ Academic Self-perception (Dimension D3)

The score found revealed that the first, fourth, and ninth periods have a positive outlook on academic outcomes (23.88, 22.51 and 21, respectively). The twelfth period revealed a negative perception (“many negative aspects” - 12.16). The mean of the four groups was 19.88, indicating a more positive perception of learning (P-value <0.05) ( Figure 1).

### Students’ Perception of Atmosphere (Dimension D4)

The first, fourth, and ninth periods revealed a positive outlook for the overall environment (“a good feeling overall” - 39.29 and “a more positive attitude” - 35.88 and 34, respectively). The twelfth period revealed a negative view of the general environment (“there are many issues that need chanching” - 24.6). The mean score of the four groups was 33.44, revealing a positive outlook. (“A more positive attitude”) (P-value <0.05) ( Figure 1).

### Students’ Social Self-perceptions (Dimension D5)

The four groups surveyed revealed a negative view in the environment of social relations (“not a nice place”), with a mean score of 21.04 (P-value <0.05) ( Figure 1).

Interpretation according to McAleer and Roff’s practical guide to using DREEM.

### Operational difficulties

The application of DREEM alone, in a pilot study conducted before this research, estimated the application time in 30 minutes. However, the DREEM statements and the questions on the sociodemographic questionnaire led to dialogues regarding the topic of medical education between the participants and the researcher after the application, which promoted a significant increase in the time of application of the questionnaires for up to 90 minutes in all groups researched. In a possible replication from the study, it is suggested to estimate the application of the questionnaires in one hour.

## Discussion

There was difficulty in understanding the Likert-type scale in the first period. The others did not present this difficulty.

White self-professed students perceived the educational environment more negatively than those who called themselves blacks, browns, Indians, or Easterners. The difference in perception of EE among university students according to their self-declared color was statistically significant, which is in line with studies such as de Machado and Barcelos ( Machado and Barcelos, 2001), which show differences in the patterns of interest and sociability between blacks and whites at a level higher, especially in the items related to students’ perceptions of teachers, their academic self-perception and the environment in general.

White self-reported students appear to be stricter in their EE assessments than self-declared nonwhite students, which matches with literature data that refer to current discussions about the relation of racial and social dimensions and EE in the public university ( Machado and Barcelos, 2001; Cunha, 2015). In Brazil, although more than half of the population is made up of blacks and browns, universities, especially public ones, have always been a stronghold almost exclusively of whites ( De Castro SOC, Almeida FM, Pereira RM, Marques HR, 2017; Oliven *et al.*, 2017). Less financial resources used by the student in his or her own livelihood are related to a lower quality of life and a more negative view of the educational environment ( Chazan, Campos and Portugal, 2015).

Studies on the health and gender professions show that there are important variations in the way in which people of different sexes structure their respective careers since graduation in Medicine. Female students seem to face greater obstacles, feel more discrimination and devaluation of their work than male students, needing to invest more effort to prove their competence. This characteristic was observed in the present study, in which female students perceived EE more negatively than male students ( Machado MH (Org.), 1995; Gonçalves and Benevides-Pereira, 2002; Vallilo *et al.*, 2011).

Higher age students, who correspond to the most advanced periods in the course, such as those who view EE more negatively than those who have just entered the course and who are still organizing the logistics for the minimum six years required for to become doctors ( Ryan RM, Connell JP, 1985). Students in the initial (first and fourth) periods seem to benefit more from the psychopedagogical support networks deployed at the institution than the final (ninth and twelfth) periods, and these networks are fundamental to the better quality of medical school and better teaching-learning ( Ryan RM, Connell JP, 1985; Yiu, 2005).

In the comparative analysis between the four groups, there is a decrease in the perception score as the student moves from the basic cycle (first period), through the clinical cycle (fourth and ninth periods) to the boarding school (twelfth period). The difference of perception between the students of the first period, who consider the excellent EE (“excellent” - 152/200) and the students of the twelfth period, near the end of the medical graduation, EE (“plenty of problems” - 99.4/200). As students attend medical school, they perceive with greater clarity the educational environment in which they are enrolled, in addition to being more critical of their needs as students ( Trindade and Vieira, 2009; Fazendeiro, 2011; Guimarães AC, Falbo GH, Menezes T, 2015).

Recent studies demonstrate that the DREEM questionnaire, when applied in AE whose structure is student-centered, has high final scores ( Al-Ayed and Sheik, 2008). This is probably due to the fact that active learning methodologies incorporate positive aspects that are contemplated in the DREEM and that are absent from the traditional teaching model used in the research institution ( Berbel, 1995; Grosseman and Stoll, 2008; Riquelme *et al.*, 2009). In the final phase of the medical course, during the internship, the student is faced with greater difficulties regarding the sense of security and faces the reality of the profession he has chosen, that is, attending to his patients ( Fabichak C, 2014; Pacheco *et al.*, 2017). The traditional teaching model based on the disease-centered Flexnerian strand does not adequately prepare the learner for the greater challenges and difficulties he will encounter after he graduates ( Al-Ayed and Sheik, 2008; Aghamolaei and Fazel, 2010; Kiran and Gowdappa, 2013).

Lack of motivation, dissatisfaction with the course, decreased academic effectiveness and lack of emotional support are important factors for the development of mental health problems in medical students, corroborating data revealed by the DREEM in the research institution.

A worrying fact revealed by DREEM relates to the student’s satisfaction in attending Medicine. The score of the affirmation “satisfaction is greater than the stress of studying medicine” decreases considerably throughout the course, being considered as a strong point of teaching for the first period (3.5), an aspect to be improved by the fourth period (2.9) and for the ninth period (2.5), and a problematic educational environment area requiring intervention by the twelfth period (1.7).

Aspects to be improved were present in all dimensions evaluated considering the score of the four periods participating in the research, offering a clear view of areas of the educational environment where intervention is needed.

About students’ perceptions of learning, the DREEM revealed a discouraging, teacher-centered and less cohesive teaching. In the perception of teachers, the DREEM revealed a lack of constructive criticism, clear examples and lack of feedback from teachers. According to the guidelines of the World Federation for Medical Education (WFME), conducting an evaluation of the educational environment considering student feedback is fundamental for medical schools (42,47). To achieve satisfaction goals, a regular program of evaluation and monitoring of possible changes is needed ( Pololi L, Frankel R, 1996).

In students’ academic self-perception, students reveal that they do not feel prepared or confident to practice the medical profession. The students’ perception of atmosphere has a significant influence on motivation and academic performance ( Genn, 2001a). Among the aspects that can be improved in this dimension, the students highlighted the lack of tranquility of the environment during the classes and in the practice places of the clinics and the infirmaries. Another aspect identified as a problem area was the capacity for concentration, a result demonstrated in other studies ( Bassaw *et al.*, 2003; Al-Hazimi, Al-Hyiani and Roff, 2004; Till, 2005), which evidences the need to reduce the volume of information in the curricula of medical schools. However, one study pointed out that students recognize the great amount of theoretical content as an inherent fact in the medical course and the continuous search for updating as fundamental throughout the career ( Whittle, Whelan and Murdoch-Eaton, 2007), which indicates that this difficulty of concentration with a common aspect to the medical curriculum in general and highlights the need to reduce the volume of information in medical school curricula ( Bassaw *et al.*, 2003; Al-Hazimi, Al-Hyiani and Roff, 2004; Till, 2005).

The students’ social self-perceptionsby the students of the four periods surveyed indicated that they consider the teaching environment to be negative (“not a nice place”), that they feel tired to enjoy the course, discouraged, alone and refer that life is not good. The absence of a program to support stressed students was highlighted as a negative factor. In environments where social relations are favorable, students are more confident and responsible ( Genn, 2001a, 2001b). However, studies point to social issues related to the cultural context of the studied population, as an explanation for the negative perception of social relations ( Mayya and Roff, 2004).

## Conclusion

The current medical education system is below an Educational Environment conducive to teaching and learning. The DREEM proved to be an efficient instrument to reveal students’ perceptions of this AE in the medical course researched. Its application and analysis revealed strengths that need greater appreciation, weaknesses that require intervention, and, mainly, students’ dissatisfaction and frustration with the traditional Flexner model of teacher-centered teaching. These points can be prioritized in curricular reforms and changes in the material and affective elements that involve and permeate the learner in order to modify and improve the educational environment. Groups that suffer social risk, whether by race or social class discrimination, tend to evaluate the educational environment more positively (or less negative) than other groups studied.

Important components of the educational environment may not have been evaluated in this study, since the DREEM instrument is an objective tool with closed questions. A study with a qualitative method associated with DREEM, such as the interview of the participating students, could allow access to unanswered questions, such as the causality of factors that benefit or hinder the teaching-learning relationship in the research institution. On the other hand, even with the limitations pointed out, the study in question brings useful subsidies to medical education.

These results demonstrate the need to take actions that make medical education less tiring and more stimulating, as well as creating environments that favor the student-centered learning process. The critical points identified in this research demonstrate the need to see the medical student as an active being in the teaching-learning process and in the educational environment. Likewise, they are a contribution to the educational institution in the sense of discerning possibilities of building a medical school that, through education, will form doctors who can transform, for the better, the universe in which they are inserted.

## Take Home Messages

• The curriculum implemented in the medical teaching institution does not define the Educational Environment, however, as well as the faculty and student body, is one of the fundamental factors for the appropriate teaching-learning environment.
• Groups that suffer social risk, whether by race or social class discrimination, tend to evaluate the educational environment more positively (or less negative) than other groups studied.
• Actions that make medical education less tiring and more stimulating favor the student-centered learning process. It is essential that teachers are committed to the improvement of medical education.

## Notes On Contributors

- This article is written from the master’s dissertation of the author Professor Débora Alves dos Santos Fernandes, who has as advisors the teachers Stella Regina Taquette and Nádia Cristina Pinheiro Rodrigues, in the Postgraduate in Medical Sciences, Faculty of Medical Sciences, of the University of the State of Rio de Janeiro (UERJ), Brazil.

- Fernandes is a Professor at the School of Medicine and Surgery of the Federal University of the State of Rio de Janeiro (UNIRIO) in Brazil. He holds a Master’s Degree in Medical Sciences from the State University of Rio de Janeiro in the thematic area of Information and Education in Health and has a degree in Medicine from UNIRIO. He has been working with the subject of Medical Education since graduation.

- Taquette is an Associate Professor at the State University of Rio de Janeiro (UERJ), Procientista, Member of the permanent body of the Graduate Program in Medical Sciences (PGCM) and the Postgraduate Program in Bioethics, Applied Ethics and Collective Health ( PPGBIOS) and Scientist of Our State by FAPERJ. She is an institutional and medical examiner at INEP-MEC-Brasil. He holds a Post-doctorate in Public Health from the Oswaldo Cruz Foundation, a Doctorate in Medicine (Child and Adolescent Health) from the University of São Paulo, a Specialization in Applied Ethics and Bioethics from the Oswaldo Cruz Foundation, a Pediatric Medical Residency and a PhD in Medicine by UERJ.

- Rodrigues is a researcher at the Sérgio Arouca National School of Public Health (ENSP) of the Oswaldo Cruz Foundation (FIOCRUZ), associate professor of Epidemiology and Biostatistics at the Faculty of Medical Sciences of the State University of Rio de Janeiro and member of the Graduate Programs. in Public Health (PGSP / ENSP / FIOCRUZ), Medical Sciences (PGCM / FCM / UERJ) and Telemedicine and Telehealth (MPTT / UERJ). He holds a Doctorate and Master’s Degree in Collective Health from UERJ, and a degree in Dentistry from Oswaldo Aranha Foundation University, UNIFOA.

## Appendices

There are no appendices.
