# Supplementary material for: The educational environment of a traditional public school of medicine in Brazil with the DREEM questionnaire
Source: MedEdPublish (2016). 2019 May 2;8:101. [Version 1] doi: 10.15694/mep.2019.000101.1 (PMC10712488; doi:10.15694/mep.2019.000101.1)
Supplement: Peer review history

# Reviewer report: Reviewer response for version 1

**DOI:** 10.21956/mep.19824.r29862

**Published:** 2019-05-24

**Authors:** Saee Deshpande

**Affiliations:** 1 Maharashtra University of Health Sciences, Nashik- VSPM Dental College Nagpur

This review has been migrated. The reviewer awarded 3 stars out of 5 The authors have taken up a very important aspect of educational environment and explored it in depth. However, language could have been more precise and with minimum grammatical mistakes. Whether questionnaire was administered face to face or electronically needs to be mentioned. Also, the point of - Groups that suffer social risk, whether by race or social class discrimination, tend to evaluate the educational environment more positively (or less negative) than other groups studied needs elaboration in discussion as well. Overall authors efforts need to be congratulated.

Reviewer Expertise:

NA

No decision status is available

# Reviewer report: Reviewer response for version 1

**DOI:** 10.21956/mep.19824.r29863

**Published:** 2019-05-10

**Authors:** Leila Niemi-Murola

**Affiliations:** 1 University of Helsinki

This review has been migrated. The reviewer awarded 3 stars out of 5 Thank you for the opportunity to review this interesting article about educational environment of a traditional medical school in Brazil. Perception of educational environment might be affected both by the educational system of the medical school and the student’s ethnic group. The former background factor is very interesting in this setting, after series of educational reforms. There is evidence of student distress and depression in several countries, but the Brazilian suicide rates sound alarmingly high.The authors have used DREEM as the instrument and it has been previously validated in Brazil – in Portuguese? The presentation of the study population is a bit unclear, because the authors refer to 1st to 12th grade students, the grades being equal to terms in this context. The heading of Table 2 does not explain all the abbreviations in the table (e.g. SiSU - ENEM), which makes the interpretation of the results a bit challenging. One feels rather sad when reading the results, especially the ones about the teaching environment. Amount of teachers’ pedagogical education would have been an interesting background factor. It would have been interesting to compare results in other medical schools having traditional curricula. Have the authors considered making the same study in some other Brazilian medical school, too?

Reviewer Expertise:

NA

No decision status is available

# Reviewer report: Reviewer response for version 1

**DOI:** 10.21956/mep.19824.r29864

**Published:** 2019-05-03

**Authors:** BALAJI ARUMUGAM

**Affiliations:** 1 TAGORE MEDICAL COLLEGE AND HOSPITAL

This review has been migrated. The reviewer awarded 4 stars out of 5 Thank you so much for inviting me to review this article. This is a very interesting article which has explored the important area of a medical graduate for whom the educational environment paves the way to learn a lot during the course. Educational environment does not only include the academic class hours, clinical case discussion, but it includes the all the physical and social environment that the medical graduate is obliged to stay during the medical school period. The objective of the study was clear and the study aimed o analyze the educational environment of a medical school and to investigate the presence of association between the sociodemographic variables of the students and their perceptions about the EE using a Standardized questionnaire (DREEM). It is an instrument originally designed to be a universal, applied in various parts of the world, and serves as a basis for the development of more particularized instruments. Its good that this study has utilized the apt valid questionnaire and was acceptable by the study participants. Students’ Perceptions of Learning , Perceptions of Teachers, Academic Self-perception, Perception of Atmosphere, Social Self-perceptions were compared among the group and among the different period. And the article concluded with low-income, self-declared non-white and female students experience more problems and negative situations in the medical school. That teacher-centered teaching is an unfavorable factor in EE in this study. I think this study will be an eye opener for many universities to conduct such large scale studies to assess the educational environment and the associated factors pertaining to their situations. GOOD JOB AUTHORS.

Reviewer Expertise:

NA

No decision status is available
